# In Vitro Release of Anthocyanins from Microencapsulated Natal Plum (*Carissa macrocarpa*) Phenolic Extract in Alginate/Psyllium Mucilage Beads

**DOI:** 10.3390/foods11172550

**Published:** 2022-08-23

**Authors:** Faith Seke, Vimbainashe E. Manhivi, Retha M. Slabbert, Yasmina Sultanbawa, Dharini Sivakumar

**Affiliations:** 1 Department of Horticulture, Tshwane University of Technology, Pretoria West 0001, South Africa; 2 Phytochemical Food Network Group, Department of Crop Sciences, Tshwane University of Technology, Pretoria West 0001, South Africa; 3 Australian Research Council Industrial Transformation Training Centre for Uniquely Australian Foods, Queensland Alliance for Agriculture and Food Innovation, Centre for Food Science and Nutrition, The University of Queensland, St Lucia, QLD 4069, Australia

**Keywords:** indigenous fruits, anthocyanins, sodium alginate, encapsulation, microstructure, thermal stability, release profile, bioaccessibility

## Abstract

Natal plum (*Carissa macrocarpa*) contains anthocyanins, cyanidin 3-*O*-β-sambubioside (Cy-3-Sa), and cyanidin 3-*O*-glucoside (Cy-3-G) that possess great bioactive properties. During in vitro gastrointestinal digestion, Cy-3-Sa and Cy-3-G are highly sensitive to pH changes and have low bioaccessibility rates of 7.9% and 22%, respectively. This study aimed to therefore use microencapsulation techniques to improve the bioaccessibility of Cy-3-Sa and Cy-3-G. The crude anthocyanin-rich extract was extracted from freeze-dried Natal plum fruit using ultrasonic-assisted ethanol extraction. The anthocyanin-rich extract was encapsulated using the ionic gelation method. Four distinct carrier agents, namely sodium alginate, pectin, xanthan gum and psyllium mucilage were used to form the wall materials. Encapsulation efficiency was highest for alginate/psyllium mucilage beads (93.67%), while alginate showed the least efficiency (86.80%). Scanning Electron Microscopy revealed a cracked and porous structure for the Natal plum extract and a continuous smooth structure for all the beads. Fourier transform infrared spectroscopy showed peaks at 3300 and 1610 cm^−1^, confirming the presence of polyphenols and polysaccharides in all beads. Thermal stability was higher for the alginate/psyllium mucilage beads and the observed thermal transitions were due to the bonds formed between the polymers and the polyphenols. Alginate beads combined with xanthan gum, pectin, and psyllium mucilage showed a prolonged release of anthocyanins compared to alginate in vitro alone. The highest anthocyanin bioaccessibility was obtained from alginate/psyllium mucilage beads (85.42 ± 1.03%). The results showed the effectiveness of alginate/psyllium mucilage beads in improving stability and in vitro anthocyanin release.

## 1. Introduction

In the food industry, antioxidants and pigments produced from natural sources are valuable additives. The stability of antioxidants and pigments (anthocyanins) is affected by their low solubility, light, pH, oxygen, and temperature of the environment, and they are rapidly destroyed during gastrointestinal digestion, resulting in lower bioaccessibility and bioactivity [1]. Designing novel oral delivery methods is therefore essential to solve this deficiency; the new system should permit control of the release of antioxidant compounds, maintain the necessary concentration, and enhance the efficacy of current formulations [2]. Microencapsulation is a technique that can be used to protect bioactive molecules from instability, while also allowing them to be used in new ways. Microencapsulation techniques include ionic gelation, spray drying, coacervation, freeze-drying, and emulsions, to name a few [2]. Having a delivery system with distinct physicochemical properties is vital to fulfilling the purpose of encapsulation, the wall materials used, and the end uses. In food and pharmaceutical applications, several natural polymers, such as alginate, have been proposed as encapsulating agents for a variety of active compounds [3]. Alginate, a food-grade copolymer, is a commonly used material for the encapsulation of many bioactive compounds due to its cost-effectiveness and compatibility [4]. Alginate is widely used to form particles for food applications because of its excellent gelling properties in low-pressure environments, making it suitable for heat-sensitive molecules. Calcium binds to the guluronate moieties of the alginate polymer chains when it interacts with it and an egg-box structure is formed [5]. Alginate gel particles, on the other hand, have varied porosity and permeability depending on alginate characteristics and processing conditions, resulting in different molecular diffusion kinetics and potential limitations in their application in food items [6]. To lessen the porosity and dispersion of the beads, combined approaches of ionic gelation and complexation with cationic polyelectrolytes have been proposed. The anionic charges of the polysaccharides and the cationic charges of the polymers interact electrostatically to cause the complexation [7]. In general, electrostatic compounds can be reversed depending on the ionic strength and pH. By interacting electrostatically with the anionic charges on the surfaces of the gel particles, substances such as hydrocolloids and proteins can offer the encapsulated compounds more protection by blocking the passage of the hydrophilic chemicals through the pores of the gel matrix. Combining alginate with proteins or polysaccharides to act as either wall material or filling agents can overcome the porosity and permeability limitations of alginate gels [7]. It has been reported that sodium alginate can form strong complexes with other natural polyelectrolytes, such as pectin, xanthan gum, and various types of mucilage (psyllium mucilage), through chain–chain association, thereby improving the mechanical and chemical stability of pure alginate beads and, as a result, the effectiveness of encapsulation [7]. Furthermore, there is no information available on the possible combined application of alginate, psyllium husk mucilage, xanthan gum, and pectin as a bioactive chemical encapsulating agent.

In addition, at a lower pH, the flavylium cation formed enables the anthocyanin (pigments) to be highly soluble in water [1], and anthocyanin-gum polymerization could possibly increase anthocyanin stability at a lower pH [8]. It has been reported that the anthocyanin molecule becomes protonated when combined with the protonated amino groups of gums, such as psyllium mucilage via ionic gelation, thereby creating a stronger barrier that reduces anthocyanin loss. Fernandes et al. [8] reported that anthocyanins provide a coating of the pectin’s surface through the development of very weak interactions, such as hydrogen bonds and van der Waals bonds. However, for hydrophilic substances, such as anthocyanin pigments, the ionic gelation process is still difficult due to issues with encapsulation effectiveness, compound diffusion, interactions between polymers and hydrophilic actives, and controlled release qualities of the core substance. Hence, it is of paramount importance to develop anthocyanin delivery systems that are more effective and facilitate targeted release. We would like to believe that this is the first description of the ionic gelation-based encapsulation of anthocyanins from *Carissa macrocarpa* (Natal plum).

*Carissa macrocarpa* (Natal plum) is an indigenous red berry fruit rich in anthocyanins, native to Natal, South Africa [9]. Natal plum contains cyanidin derivatives cyanidin 3-*O*-β-sambubioside (Cy-3-Sa), cyanidin 3-*O*-pyranoside, and cyanidin 3-*O*-glucoside (Cy-3-g) [9,10]. The fruit extract also has interesting, potentially health-promoting benefits, including antioxidant and α-glucosidase inhibitory activities. Seke et al. [9] demonstrated that during simulated gastrointestinal digestion the anthocyanins from the Natal plum were highly unstable with low bioaccessibility [9]. The low bioaccessibility of Natal plum anthocyanins can hinder their functionality in the body. The efficiency of anthocyanins and polyphenols depends on their integrity and bioaccessibility [1]. The action of anthocyanins and polyphenols and their potential therapeutic benefits are constrained by their unsteadiness and brittle stability during food preparation, circulation, or in the gastrointestinal tract [9]. Furthermore, they degrade quickly due to rapid oxidation, which could hinder the effectiveness of using these polyphenols in the pharmaceutical and nutraceutical industries. Hence it is necessary to introduce microencapsulation to protect the bioactive compounds from adverse conditions during gastrointestinal digestion, such as unfavorable pH changes. Apart from the health benefits, beads containing polyphenol (anthocyanin) extracts can be used as functional additives and preservatives in the formulation of intricate food systems and to prolong the shelf life of foods [11]. 

This study aimed to investigate the encapsulation of Natal plum anthocyanin extract in different delivery systems (alginate, alginate-pectin, alginate-xanthan gum, and alginate-psyllium mucilage). Encapsulation efficiency was used to assess the process’ efficiency, while polymer–polyphenol interactions, microstructure, and thermal stability were also assessed. The recovery, bioaccessibility, and release behavior of the anthocyanin extract in vitro was also assessed.

## 2. Materials and Methods

### 2.1. Reagents and Standards

A commercially available, food grade *Plantago psyllium* husk, sodium alginate (pure (>99%) food grade, viscosity 15–25 cp), porcine bile, xanthan gum (XG, viscosity; 1785 cps), citrus pectin with a high degree of esterification (53%), sodium acetate (≥99%), sodium citrate (99.5), sodium chloride (≥99%), potassium phosphate (≥98%), acetate buffer (pH 5.2 ± 0.1 (25 °C)), α-Amylase from porcine pancreas, potassium chloride (≥99%), pancreatin from porcine pancreas (8 × USP), pepsin (powder, ≥250 units/mg solid), magnesium chloride (anhydrous, ≥98%), bovine bile (dried, unfractionated, NA.85), HCl (ACS reagent, 37%), ferric chloride (ACS reagent, 37%), ethanol (95%), calcium chloride (99%), ammonium carbonate (ACS reagent, ≥30.0% NH3 basis), were procured from Sigma Aldrich (Johannesburg, South Africa). All other reagents used were of analytical grade purity. 

### 2.2. Natal Plum Phenolic Extraction and Alginate Beads Preparation 

**Figure 1:**
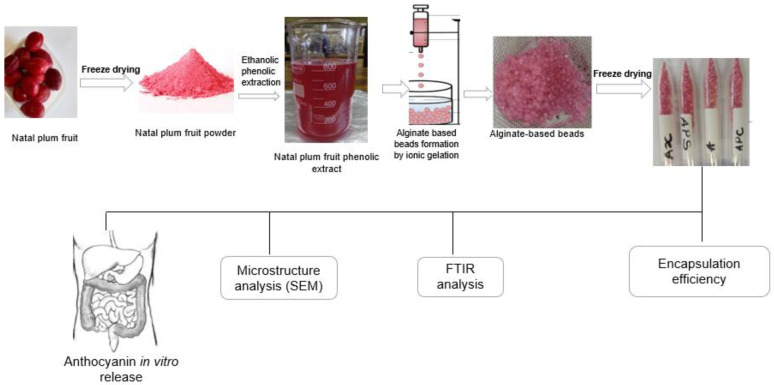
Schematic representation of the development of alginate-based beads and analysis.

Natal plum phenolic extract was produced using a previously described method by Ndou et al. [10]. Natal plum sample (10 g) was extracted using 80:20 ethanol/water (100 mL) and was ultrasonicated for 30 min at 30 °C. The supernatant was obtained by centrifugation at 3000× *g* for 20 min at 4 °C using a Hermle Z326k, Hermle Labortechnik GmbH, Wehingen, Germany). The supernatant was collected. Alginate beads were made following a method described by Li et al. [12]. Alginate (2% *w*/*v*, 2 g/100 mL), alginate (2% *w*/*v*) in combination with xanthan gum (2% *w*/*v*, 2 g/100 mL), or pectin (2% *w*/*v*, 2 g/100 mL) or psyllium mucilage (1% *w*/*v*, 1 g/100 mL) was dispersed in distilled water and agitated until completely dispersed. The alginate mixture was dropped into a Natal plum extract solution (0.5% *w*/*v*) containing calcium chloride solution (5% *w*/*v*), using a syringe under manual control. Calcium chloride was used as a hardening solution. Beads were allowed to sit in the hardening solution for 10 min to ensure complete hardening. The filtrate was then recovered after the beads had been filtered through a Whatman #4 paper filter. After that, water was used to wash the beads. The schematic representation of the process is illustrated in Figure 1.

### 2.3. Total Anthocyanin Content and Encapsulation Efficiency

The total anthocyanin content of the beads was determined using a method reported by Aizpurua-Olaizola et al. [13]. Beads structures were destabilized by homogenizing with 5% sodium citrate (10 mL) with constant stirring until complete dissolution had been achieved. The total anthocyanin content (TAC) was measured using a pH differential method based on the structural changes in chemical forms of anthocyanin, as described by Seke et al. [9]. The Natal plum extract (1.5 mL) was mixed with 2.5 mL 0.025 M potassium chloride (pH 1) or 2.5 mL of 1 M sodium acetate (pH 4.5). The color eluted by anthocyanin is pH-dependent [9]. At pH 1.0 the molecule is pigmented, and it is colorless at a pH ≥ 4.5. Therefore, anthocyanins (pH, 1.0) absorb light more between 460 and 550 nm and colorless at a pH of 4.5. It is possible to detect the total anthocyanins accurately and quickly even in the presence of polymerized deteriorated pigments and other interfering compounds since the difference in the pigment’s absorbance at 520 nm is proportional to the concentration of pigment [13]. The solution was incubated for 30 min in the dark, and the absorbance was measured at 520 nm and 700 nm (EZ Read 2000; Biocrom Ltd., Cambridge, UK). TAC was expressed as mg equivalents of cyanidin-3-glucoside per g dry weight basis. The encapsulation efficiency was calculated using Equation (1), described by Mendes et al. [14], as follows.

(1) $$\mathrm{EE}\%=\frac{\mathrm{Anthocyanin}\;\mathrm{content}\;\mathrm{in}\;\mathrm{beads}}{\mathrm{Anthocyanin}\;\mathrm{content}\;\mathrm{in}\;\mathrm{the}\;\mathrm{extract}}\;\times \;100\;$$

### 2.4. Microstructure of the Beads

The microstructure of freeze-dried Natal plum powder and alginate beads was examined using a JEOL Scanning Electron Detector microscope (SEM) with an Energy Dispersive X-ray, running at 3 kV [15]. The samples were mounted directly on door-metallic specimens of 12 mm in diameter and 10 mm in height and then metallized using a thin layer of gold measuring 0.1 mm in thickness of 200 Å. The samples were then observed with magnifications of 2000, 2500, 5000, and 10,000×.

### 2.5. Thermogravimetric Analysis (TGA)

The thermostability of the beads was analyzed using a method by Mendes et al. [14]. To measure the loading efficiency of bioactive compounds, a TGA Q500 analyzer (TA Instruments-Waters LLC, New Castle, DE, USA) was employed with the temperature set to 900 °C and a heat rate of 10 °C min^−1^ in nitrogen.

### 2.6. Fourier Transform Infrared Spectroscopy (FT-IR) 

Polymer–polyphenol interactions in anthocyanin-enriched freeze-dried beads were investigated using Fourier transform infrared spectroscopy (FT-IR), following a methodology proposed by Fathordoobady et al. [15]. Potassium bromide was added to alginate-based beads and then mixed in a mortar with a pestle and pressed into pellets. An FT-IR spectrometer (Perkin Elmer Spectrum 100 spectrometer, Waltham, MA, USA) was used to capture infrared spectra in the wavelength range 600–4000 cm^−1^. A total of 32 scans were done and spectra resolution was maintained at 4 cm^−1^.

### 2.7. In Vitro Release Behavior of Anthocyanins

The in vitro release properties of the Natal plum anthocyanins were done following a method by Marefati et al. [16]. A total of 10 mL salivary fluid was added to the Natal plum extracts and agitated at 170 rpm. After 2 min of continuous agitation, gastric fluid (20 mL) was added to the mixture and the pH was adjusted to 2.5. The slurries were then incubated for 2 h at 37 °C, then a 10 mL aliquot was collected to stop enzymatic reactions and then stored at −80 °C for further analysis. Simulated intestinal fluid (SIF) (20 mL) was then added and, adjusting the pH to 7.5, the digesta was incubated for 2 h. The digesta were stored at −80 °C after digestion for future analysis. Percentage recovery after gastric phase and bioaccessibility after intestinal phase was calculated using Equations (2) and (3), respectively. Additionally, after specific time intervals, aliquots (2 mL) of the reaction solution were taken (10, 30, 60, 90, and 120 min) and the percentage of release under simulated gastric and intestinal conditions was calculated.

(2) $$\%\;\mathrm{Recovery}=(\frac{B_{GC}\;}{B_{ND}})\times 100$$

The *B_GC_* (TAC mg C3G g^−1^ DW)-was the phenolic compounds content in the gastric digesta and *B_ND_* (TAC mg C3G g^−1^ DW) was the anthocyanin content in the undigested beads.

(3) $$\mathrm{Bioaccessibility}\;\%=(\frac{B_{SI}\;}{B_{ND}})\times 100$$

The *B_SI_* (TAC mg C3G g^−1^ DW) was the phenolic compound content in the intestinal digesta and *B_ND_* (TAC mg C3G g^−1^ DW) was the anthocyanin content in the undigested beads.

The color of the beads during in vitro digestion was measured using a Minolta CR-400 chromameter (Minolta, Osaka, Japan). The brightness was represented by the L* value, positive a* values represented the intensity of the red color.

### 2.8. Statistical Analysis

All of the analyses were done in triplicate and twice. Tukey’s multiple range test and analysis of variance (ANOVA) were used to assess whether the values of *p* < 0.05 were substantially different. The data were analyzed using the statistical application Minitab for Windows (2018).

## 3. Results

### 3.1. The Effect of Different Microencapsulation Wall Materials on Encapsulation Efficiency

**Figure 2:**
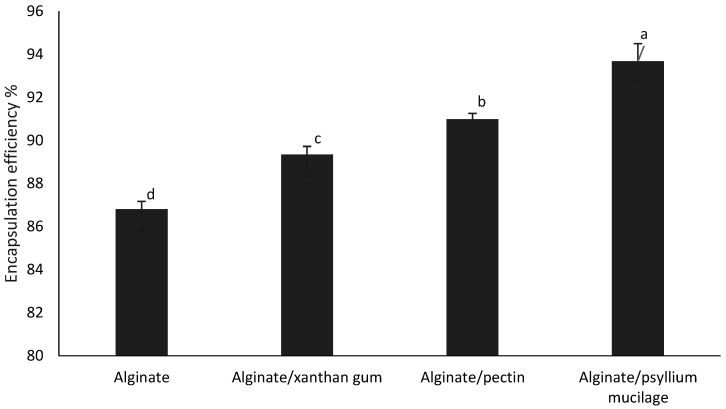
Effect of different polymers on the encapsulation efficiency of alginate-based beads. Error bars show the standard deviation value. Different letters over error bars indicate statistically significant results.

The evaluation of encapsulation efficiency is a good way to see if the applied method can entrap active materials efficiently. As seen in Figure 2, the type of polymer utilized had a substantial (*p* < 0.05) impact on encapsulation efficiency. The best encapsulation efficiency was found in alginate/psyllium mucilage beads, followed by alginate/pectin, alginate/xanthan gum, and alginate. While encapsulation efficiencies are significantly different, alginate/pectin, alginate/xanthan gum and alginate/psyllium mucilage beads were still high, the encapsulation efficiency of alginate by itself (approximately 87%) can also be considered high. These findings are consistent with previous research on polymers encapsulated in alginate and pectin hydrogels [17]. The efficiency of encapsulation was improved by adding different polymers to the alginate system. This can be attributed to the different structures of the added polysaccharides with the hydroxyl groups of the phenolics in the Natal plum extract favoring the formation of hydrogen bonds with free carboxyl end groups of the polysaccharides used [18]. Alginate is a polysaccharide composed of *β*-D-mannuronic acid and 1–4 linked *α*-L-guluronic residues, organized in a homogenous or heterogeneous pattern. The pKa is between 3.38–3.65. Xanthan gum is a polysaccharide consisting of a β-(1→4) d-glucose chain backbone, with a glucose molecule in every second position of the backbone substituted at C3, with a trisaccharide side chain consisting of β-d-mannose-(1→4)-β-d-glucuronic acid-(1→2)-α-d-mannose. The terminal mannose on the side chain is partially substituted with a pyruvate residue and acetylated at the C-6 position. Psyllium gum is a highly branched anionic glycoprotein with a high molecular weight of approximately 1500 kDa [19,20]. Conversely, pectin is a heterogeneous polysaccharide with three main structural domains, including a homogalacturonan switching with two types of highly branched rhamnogalacturonans sections. Pectin is anionic at neutral pH but approaches zero charges at low pH [21]. The pKa-value of pectin is approximately 3.5. The pH of the formulation could therefore affect the degree of ionization of the polysaccharide molecules and their electrostatic interactions with cations, such as anthocyanins. Anthocyanins, being stable in acid, are complexed at low pH and this might reduce the efficiency of encapsulation [22]. Gum Arabic was similarly also proposed to interact with tea polyphenols through its protein moiety and hence enhance the encapsulation efficiency [12].

### 3.2. Impact of Encapsulation on the Surface Morphology of the Beads

**Figure 3:**
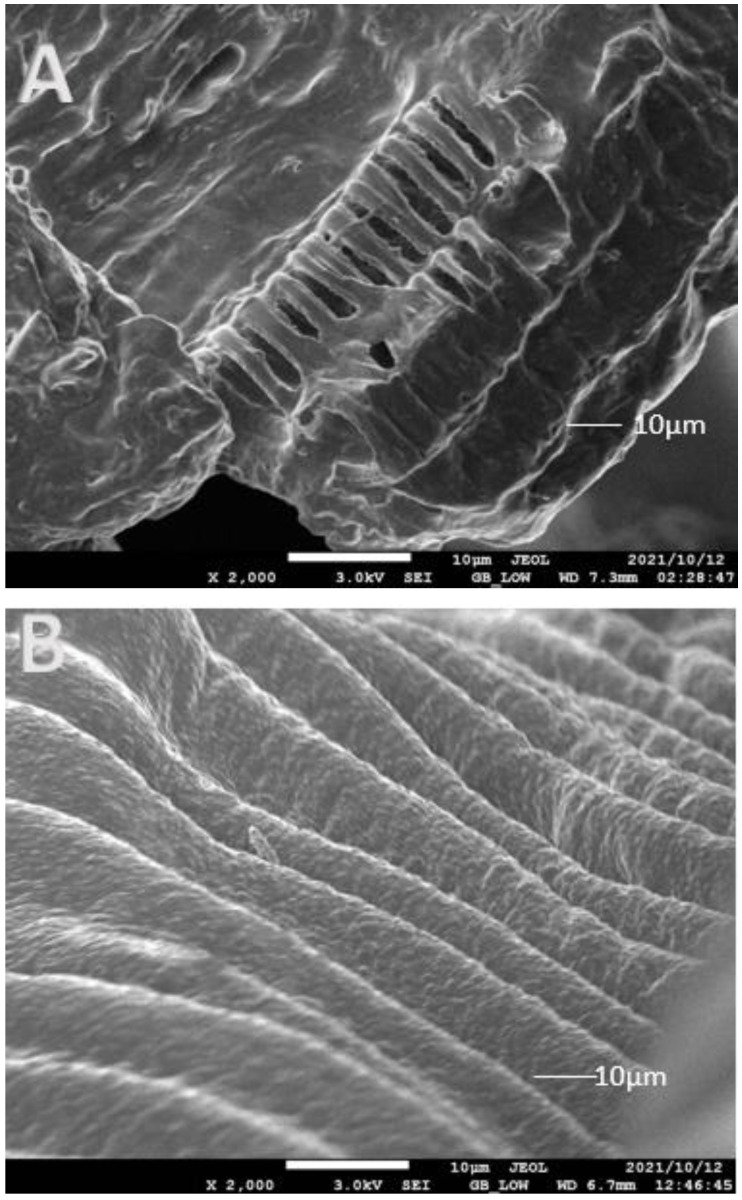

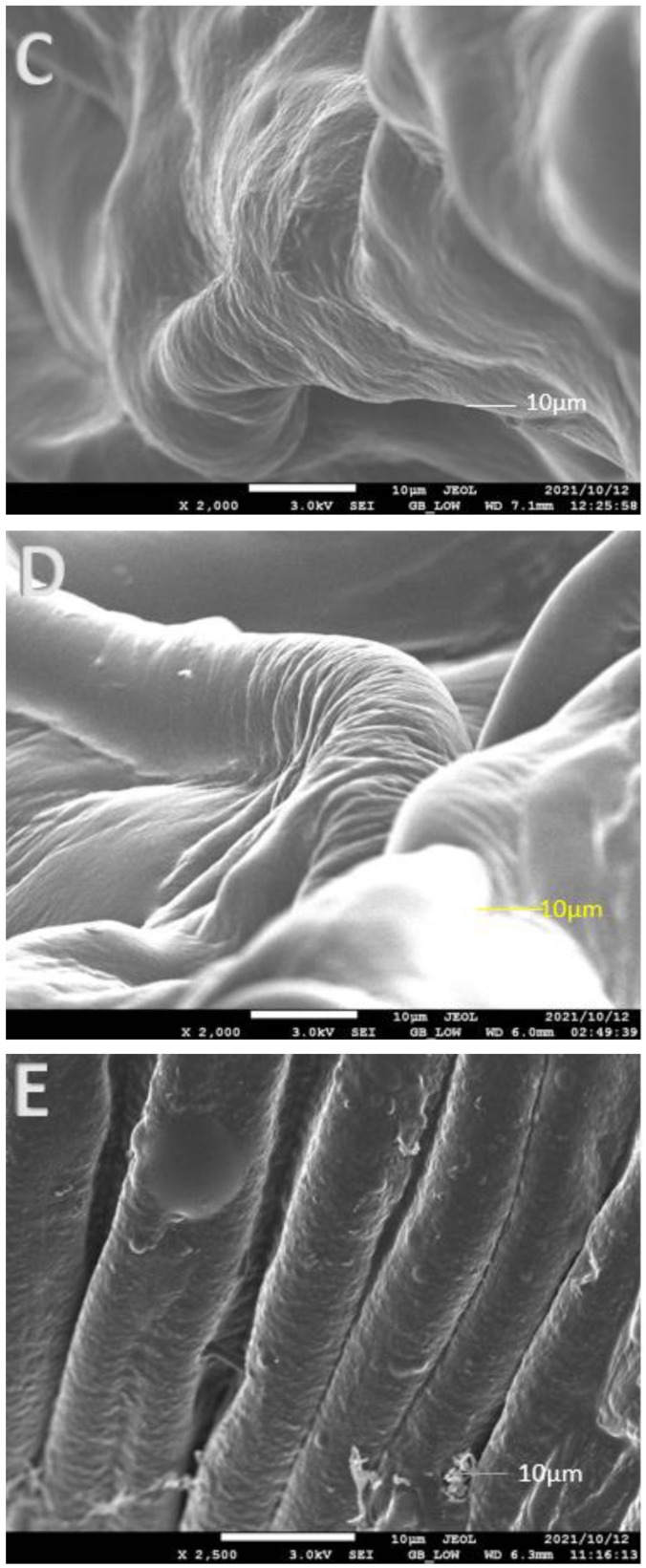
SEM microphotographs of Natal Plum enriched alginate beads and Natal plum powder. (**A**): freeze dried Natal plum powder, (**B**): alginate/psyllium mucilage, (**C**): alginate/pectin, (**D**): alginate/xanthan gum, (**E**): alginate.

SEM images of freeze-dried Natal plum fruit and beads (Figure 3A–E) allowed the surface morphology of the fruit powder and variably formed alginate beads to be characterized. The morphology of cross-linked alginate beads is altered by freeze-drying, which emphasizes irregular forms and heterogeneous surfaces. The drying steps had a significant impact on the morphology of all samples in our study, particularly those obtained with Natal plum powder, which had a cracked surface (Figure 3A). The presence of fissures in dried Natal plum powder can facilitate oxygen transfer and chemical contact, resulting in phenolic compound breakdown [23]. Encapsulation of the Natal plum extract with alginate resulted in a smooth surface with no cracks; furthermore, combining alginate with other biopolymers, such as pectin, xanthan gum and psyllium mucilage, resulted in a more homogenous morphological structure. Flamminni et al. [17] showed similar prevention of a cracked surface with a thick and continuous structure when alginate and pectin were used to encapsulate phenolic compounds in olive extract. Biopolymers added to the alginate matrix increased the gel network’s resilience during the drying process by acting as structural reinforcement against beads shrinking and collapsing [18]. The addition of psyllium mucilage to the alginate matrix resulted in a surface that was noticeably uneven, rough, and porous. Alginate, alginate/pectin, and alginate/xanthan gum all had a thick and continuous structure with a heterogeneous profile. This might be due to the formation of an interconnection chain resulting from intermolecular alginate-psyllium mucilage. 

### 3.3. Fourier Transform Infrared Spectroscopy (FTIR) Analysis 

**Figure 4:**
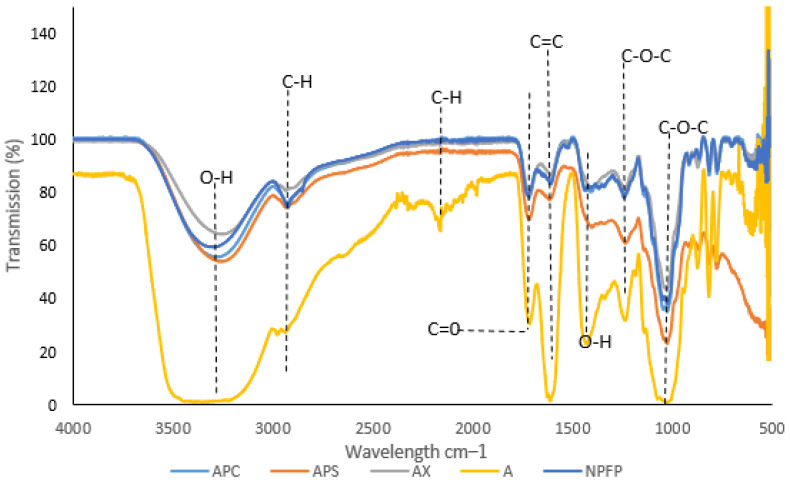
Fourier transform infrared spectroscopy measurements (FT-IR) of A-alginate, APS-alginate: psyllium mucilage, AX-alginate: xanthan gum, APC-alginate: pectin beads and NPFP-Natal plum powder.

FTIR was used to investigate the different spectra of Natal plum powder and anthocyanin-infused alginate-based beads to determine the possible molecular interactions between the elements of the four delivery systems (Figure 4). Distinct peaks at approximately 3300, 2932, 2160, 1717, 1610, 1426, 1244, and 1031 cm^−1^ confirmed that the extract’s major constituent is anthocyanins. The wide peak at 3216 cm^−1^ proposed that O–H bonds were stretching vibrationally. This may be due to intramolecular and intermolecular bonding, as well as free hydroxyl groups. Alginate beads exhibited a bigger and a broader region for O–H stretching in the presence of Ca^2+^ ions. C–H bonds were lengthening, as shown by the peak at 2932 cm^−1^. The stretching vibration of C–O at 1706 cm^−1^ and the bending vibration of C–O–C groups at 1031 cm^−1^ indicated the existence of carbohydrates. The band at 1244 cm^−1^ matched the aromatic rings’ skeletal stretching vibration and the flavonoids’ C–O–C group [24]. The bands observed in this study are comparable to those found in crude jussara fruit extract [25]. All of these functional groups confirmed the beads’ high anthocyanin content. Anthocyanins as cyanidin compounds, in particular, have an absorption spectra region of 3100–3400 cm^−1^ (O-H symmetric stretching vibration) with others showing at 2900–2840 cm^−1^ (C–H aliphatic), 675–870 cm^−1^ (C–H aromatic) and 1660 cm^−1^ (C=C aromatic), confirming the presence of phenolic compounds within the alginate beads [26]. Furthermore, bands related to the skeletal stretching vibration of aromatic rings and the C–O–C group of flavonoids (1260, 1076 and 1516 cm^−1^) were detected via several peaks that show increasing intensity on the alginate.

O-H in-plane deformation may potentially be responsible for the 1363 cm^−1^ absorption band in polyphenols. The deformation vibration of C–C bonds in phenolic groups adsorbs in the 1500–1400 cm^−1^ range [27]. The addition of different biopolymers to alginate showed that the original functional structure and the integrity of Natal plum powder were retained, and the combination of encapsulation prevented the loss of bioactive compounds.

### 3.4. The Effect of Encapsulation on the Thermal Stability of Anthocyanins in Encapsulated Alginate-Based Beads

**Figure 5:**
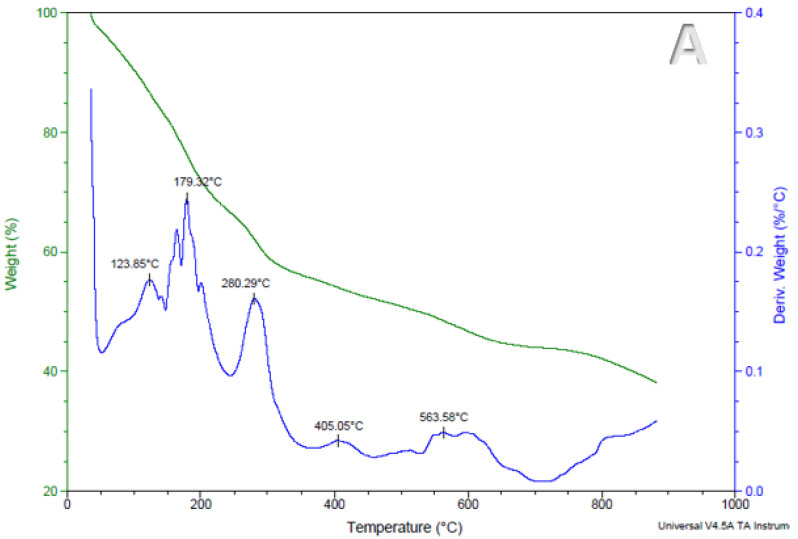

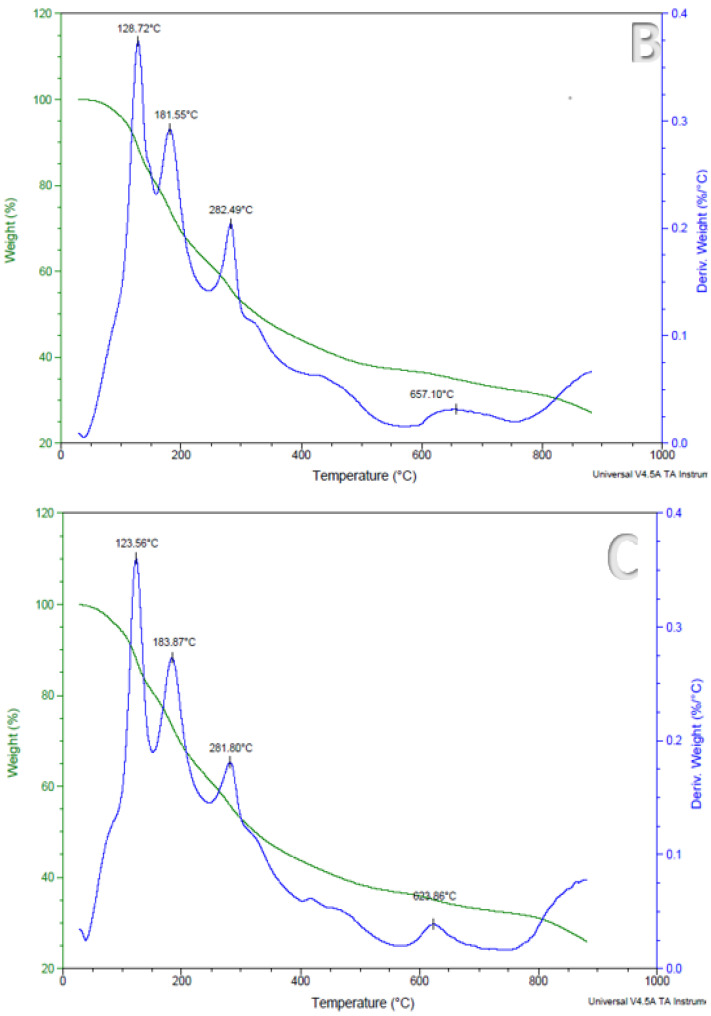

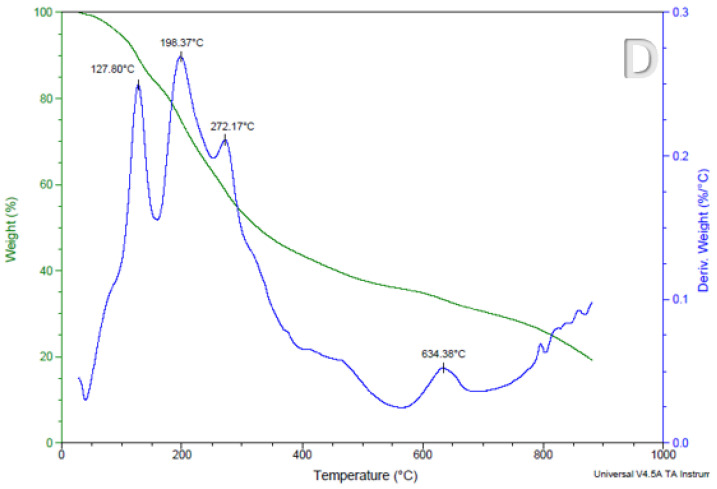
Curves of thermogravimetric analysis of treatments from alginate beads (**A**), alginate: xanthan gum beads (**B**), alginate: pectin beads (**C**) and alginate: psyllium mucilage beads (**D**).

Anthocyanin retention in foods has a significant impact on their sensory qualities, including flavor, taste, and appearance [28]. During processing anthocyanins are highly unstable therefore it is critical to investigate the accelerated thermal stability of encapsulated Natal plum anthocyanins before incorporating them into foods. The thermogravimetric analysis investigates the thermal degradation of a sample due to mass loss over a given temperature range [29]. The samples were heated from 10 °C to 900 °C, and the mass loss of the beads is represented in Figure 5A–D. The mass loss findings were consistent across all treatments, as shown in Figure 5A–D on the mass of the derivate axis represented in %/min. The evaporation of the water first absorbed by the beads, which happens between 20 and 200 °C, causes the first and second mass losses. The deterioration of biopolymers and disintegration of encapsulated material caused a significant loss of mass at approximately 280 °C. Additionally, mass loss in the 170–370 °C range could be linked to glycosidic bond obliteration [14]. The temperature range (close to 800 °C) leads to the degradation of sodium alginate [30]. The residue amounts for the alginate beads are approximately 36%, 26%, 20%, and 27% for alginate, alginate/pectin, alginate/xanthan gum and alginate/psyllium, respectively. Under nitrogen, thermal analysis of organic material results in the formation of residual carbon due to formation of non-volatile structures [31]. The differences in the residue amounts can be linked to the structures and molecular weight of polysaccharides used in bead making [32]. Furthermore, alginate on its own may have incorporated more calcium to gel and hence formed more metal oxide residues. Psyllium mucilage is a high molecular weight polysaccharide (20,000,000 Da) [33] and has higher amounts of carbonaceous material, which may also contribute the higher residual weight. Pectin has a molecular weight of between 50,000 and 180,000 Da [34]. Xanthan gum has the least molecular weight (933.7 g/mol) and hence the least residual weight. A similar thermogravimetric profile has been reported in a study on jabuticaba extracts alginate-based beads developed using different alginate concentrations [14]. These variations in thermograms between pure native polymers and beads demonstrate the existence of ionic interactions that may result in the development of novel structures with various thermal properties [30]. These findings indicate that the beads we created exhibit stability under typical physiological conditions.

### 3.5. In Vitro Release Behavior of Anthocyanins

**Figure 6:**
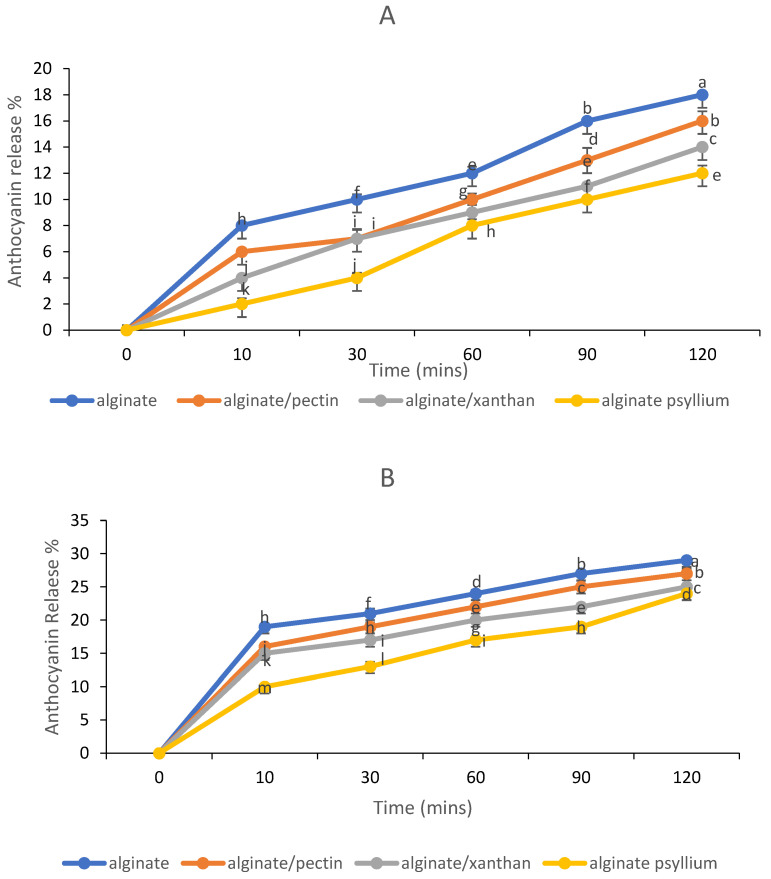
(**A**) gastric release and (**B**) intestinal release of anthocyanins from alginate beads.

The bioavailability of anthocyanins in plasma after consuming a meal is limited [35], which is often related to their low stability, permeability, solubility, and gastrointestinal tract metabolism [36]. Bioactive substance encapsulation can increase bioavailability by increasing water solubility and allowing for release in a precise environment, ensuring increased absorption by the body [37]. The ability of anthocyanin extracts encapsulated to withstand physiological gastrointestinal media for anthocyanin-loaded bead release behavior was investigated. The beads were subjected to simulated gastric fluid (pH 1.5) and simulated intestinal fluid (pH 6.8). In comparison to the other samples, the sample coated with psyllium mucilage released fewer anthocyanins in the gastric phase (Figure 6A). Throughout the 120 min of study, the release of anthocyanins increased in general. The anthocyanin release profile revealed that the porosity of alginate beads still permitted anthocyanin compounds to diffuse out of the beads. The percentage of released anthocyanins to the gastric phase in alginate beads containing extract was only 18%, while samples containing pectin, xanthan gum, and psyllium mucilage had 16, 14, and 12%, respectively. Alginate is frequently converted to insoluble alginic acid in an acidic medium [38]. The swelling of calcium alginate gels in an acidic environment may have limited the diffusion of loaded anthocyanins into the media during the first half-hour of incubation, resulting in anthocyanin release being delayed for the next 1.5 h in acidic SGF [39]. Psyllium-alginate beads delayed anthocyanin release in the simulated gastric phase, demonstrating that they were more stable in an acidic environment (pH = 1.5). After simulated stomach digestion, all of the beads in the study retained their spherical shape and red color. Kim et al. [40] suggested that the release of anthocyanin is approximately proportionate to the shrinkage of the beads, showing that squeezing pressure is the dominant dynamic factor for anthocyanin release in SGF. The thick matrix of the constricted beads may prevent anthocyanin molecules from moving, resulting in the release of anthocyanin being terminated after 2 h of incubation. 

During the intestinal phase, the integrity of the alginate beads was maintained for the first 10 min, but, after that, the amount of anthocyanin extract released from the beads increased (Figure 6B). During a 2 h incubation in SIF, the beads released some of the entrapped anthocyanins (24–29%). The release profiles of all types of alginate beads in SIF were found to be identical although alginate/psyllium beads showed a delayed-release. As can be seen, the mechanism of release in SIF differed from that of SGF. This was most probably because alginate has varying solubilities in acidic and alkaline environments. This is due to the interaction of Ca^2+^ ions associated with –COO– groups of alginates, which are primarily defined by polymannuronate sequences, with Na^+^ ions present in the saline solution at pH 6.8. [41]. Chain relaxation and gel expansion are caused by increased electrostatic repulsion between the negative charges of the –COO– groups. Because the polyguluronate sequences have significant auto-cooperation binding to calcium ions, promoting stable cross-links within the gel structure, when the calcium ions in the “egg-box” structure disintegrate and diffuse into the medium, the gel beads begin to dissolve [42]. Other mechanisms, such as those proposed in Section 3.1, could have formed and preserved alginate/psyllium beads.

### 3.6. Effect of Gastrointestinal Fluids on the Total Anthocyanin Content and Color of the Alginate-Based Beads

An in vitro digestion model was used to calculate the anthocyanin released during digestion. The total anthocyanin content of the beads followed the same pattern in all of the digestion phases (Table 1). The beads showed an anthocyanin recovery of between 83.46 ± 0.88% and 92.8 ± 1.68% after exposure to gastric juices. These results showed the positive effect of the encapsulation systems on anthocyanin stability compared to the recovery (39%) that was reported in our previous study on unencapsulated Natal plum extract [9]. Alginate beads increased anthocyanin bioaccessibility to 66.41 ± 0.45% whilst alginate/psyllium mucilage beads resulted in the highest anthocyanin bioaccessibility of 85.42 ± 1.03%. Alginate-based beads took longer to dissolve in the stomach, and under the effect of acid, they may have formed aggregates. As a result, the anthocyanins were encapsulated within an alginate matrix that was resistant to gastric digestion and considerably slowed anthocyanin diffusion. During the intestinal phase, the contents were further reduced. The decreased anthocyanin concentration in the intestinal phase could be due to the beads gradually dissolving in the neutral-alkaline solution of the intestinal fluid, causing some of the anthocyanins to leach out. Many factors affect the release of core materials from powder microparticles during in vitro digestion, including microparticle shape, powder solubility, interactions between core and wall components, and enzyme and ion resistance [43].

The L* and a* parameters of samples from gastric and intestinal fluids after 120 min are presented in Table 2. The trend that was followed by the dissolved beads was as follows: undigested < gastric phase < intestinal for the L* values indicated lower levels of whiteness in the undigested beads, which was supported by the * values that were on significantly higher levels, indicating a higher intensity of redness after 120 min. The color eluted by anthocyanin is pH-dependent [9]. At pH 1.0 the molecule is pigmented and colorless at a pH ≥ 4.5. Therefore, during gastrointestinal digestion the anthocyanins are exposed to rigorous pH changes that can affect their structure and pigmentation [13]. Hence, it is important to determine the impact of encapsulating anthocyanins using different biopolymers on the anthocyanin stability in vitro. Incorporating other biopolymers to the alginate system showed improved redness stability thereby indicating the improved stability of anthocyanin compounds in the gastric phase. After simulated intestinal digestion, alginate/psyllium beads were redder compared to all other beads. When compared to the gastric phase, the intestinal phase had a propensity to be whiter (greater L*) compared to the gastric phase. The pH levels of the intestinal phase could be the reason for this behavior. The stability of anthocyanins is greatly reliant on pH, with neutral-alkaline conditions making them unstable [9]. Furthermore, beads may disintegrate and release anthocyanins. 

## 4. Conclusions

The anthocyanin-rich phenolic extract from Natal plum fruit was encapsulated. Alginate/psyllium gum beads resulted in the most efficient encapsulation compared to beads containing alginate alone or combined with xanthan gum or pectin. The microstructural analysis showed that encapsulation led to a smooth structure with no cracks compared to the unencapsulated extract. FTIR analysis confirmed the presence of polyphenols and that the extract was successfully incorporated into the beads. Peaks at approximately 3300, 2932, 2160, 1717, 1610, 1426, 1244, and 1031 cm^−1^ confirmed that the extract’s major constituent is anthocyanins. Thermal stability analysis also revealed a positive effect of gelation on the physicochemical stability of the encapsulated Natal plum extract. Due to their increased swelling properties in bioactive compound-loaded beads at a higher pH, natural polymers significantly affect mechanical properties, porosity, and in vitro release control. As a result, combing alginate with other polysaccharides resulted in the delayed release of anthocyanins from the beads in vitro. The percentage of released anthocyanins to the gastric phase in alginate beads containing extract was only 18%, while samples containing pectin, xanthan gum, and psyllium mucilage had 16, 14, and 12%, respectively, and the beads released some of the entrapped anthocyanins (24–29%). Generally, alginate/psyllium beads can be used for food additives and for oral delivery of natal plum anthocyanins. 

## Figures and Tables

**Table 1:** Effect of gastrointestinal digestion on the anthocyanin profile of alginate-based beads.

Beads	TAC mg C3G g^−1^ DW Undigested	TAC mg C3G g^−1^ Gastric Digestion	Recovery %	TAC mg C3G g^−1^ Intestinal Digestion	Bioaccessibility %
Alginate	32.71 ^a^ ± 0.57	27.30 ^b^ ± 0.85	83.46	22.38 ^c^± 0.29	66.41
Alginate + pectin	37.22 ^a^ ± 0.31	33.29 ^b^ ± 0.20	89.44	27.92 ^c^ ± 0.89	75
Alginate + xanthan gum	36.55 ^a^ ± 0.29	30.19 ^b^ ± 0.89	82.59	24.18 ^c^ ± 0.32	68
Alginate + psyllium gum	40.14 ^a^ ± 0.18	37.29 ^b^ ± 0.37	92.89	34.29 ^c^ ± 0.76	85.42

Means followed by different letters in a row differ significantly at *p* < 0.05.

**Table 2:** Effect of gastrointestinal digestion on the color profile of alginate-based beads.

	Undigested Beads		Gastric Phase		Intestinal Phase	
	L*	a*	L*	a*	L*	a*
Alginate	2.47 ^a^ ± 0.37	12.36 ^d^ ± 0.23	4.59 ^a^ ± 0.19	8.76 ^c^ ± 0.37	16.41 ^a^ ± 0.75	1.69 ^c^ ± 0.28
Alginate + pectin	1.63 ^b^ ± 0.17	13.28 ^c^ ± 0.55	2.67 ^b^ ± 0.26	12.10 ^b^ ± 0.01	12.65 ^b^ ± 0.83	2.21 ^b^ ± 0.14
Alginate + xanthan gum	1.53 ^b^ ± 0.38	13.94 ^b^ ± 0.79	2.64 ^b^ ± 0.09	12.64 ^b^ ± 0.13	12.79 ^b^ ± 0.27	2.11 ^b^ ± 0.79
Alginate + psyllium gum	1.36 ^c^ ± 0.27	16.38 ^a^ ± 0.35	2.59 ^c^ ± 0.19	14.27 ^a^ ± 0.03	10.55 ^c^ ± 0.13	3.83 ^a^ ± 0.08

Values are Means ± SD. Means in a column followed by different letters differ significantly at *p* < 0.05.

## Data Availability

The corresponding author can provide data from this study upon request.
